# Resistive index of ophthalmic artery correlates with retinal pigment epithelial alterations on spectral domain optical coherence tomography in diabetic retinopathy

**DOI:** 10.1186/s40942-018-0116-0

**Published:** 2018-04-09

**Authors:** Manila Khatri, Sandeep Saxena, Apjit Kaur, Shashi K. Bhasker, Manoj Kumar, Carsten H. Meyer

**Affiliations:** 1 Department of Ophthalmology, King George’s Medical University, Lucknow, U.P. 226003 India; 2 Department of Radiodiagnosis, King George’s Medical University, Lucknow, U.P. India; 3 Department of Ophthalmology, Pallas Klinik, Aarau, Switzerland

**Keywords:** Resistive index, Diabetic retinopathy, Ophthalmic artery, Retinal pigment epithelium

## Abstract

**Background:**

Retinal pigment epithelium (RPE) plays a significant role in maintenance of integrity of retinal photoreceptors and choriocapillaries. RPE derives its blood supply through ophthalmic artery (OA) via choriocapillaries. RPE topographic alterations have been observed to be associated with severity of retinopathy. The present study was undertaken to assess the correlation between resistive index (RI) of the OA with RPE topographic alterations on Spectral-Domain optical coherence tomography (SD-OCT), to our knowledge, it is for the first time.

**Methods:**

A tertiary care center based cross-sectional study was undertaken after informed consent. Sample size was calculated using 95% confidence interval. Seventy five consecutive cases of type 2 diabetes mellitus between the ages of 40 and 70 years were included. The cases were divided into three groups according to Early Treatment Diabetic Retinopathy Study classification: diabetes mellitus with no retinopathy (No DR) (n = 24); non-proliferative diabetic retinopathy (n = 27); and proliferative diabetic retinopathy (n = 24). Healthy control subjects of similar age group were included (n = 24). RI in OA was studied using Color Doppler imaging. Grades of RPE topographic alterations and retinal photoreceptor ellipsoid zone (EZ) disruption were studied using SD-OCT. Data was analysed using Chi square (χ^2^) test, analysis of variance (ANOVA), Pearson correlation analysis and Neuman–Keuls test.

**Results:**

LogMAR best corrected visual acuity was found to increase significantly with the severity of DR (F = 105.74, *p* < 0.001). ANOVA revealed a significant increase in RI of OA (F = 14.23, *p* < 0.001) with severity of diabetic retinopathy. χ^2^ test revealed significant increase in grades of RPE alterations (χ^2^ = 71.83, *p* < 0.001) and EZ disruption (χ^2^ = 60.59, *p* < 0.001) with the severity of diabetic retinopathy. Pearson correlation analyses revealed a significant positive correlation between RI of OA with grades of RPE alterations (r = 0.48, *p* < 0.001) and also between grades of RPE alterations and EZ disruption (r = 0.82, *p* < 0.001).

**Conclusions:**

Decrease in ocular blood flow resulting from an increase in RI of OA correlates with severity of DR and grades of topographic alterations in RPE. Integrity of EZ was observed to be dependent on RPE.

## Introduction

Diabetes mellitus will be the seventh leading cause of death in 2030 as projected by WHO [1].The prevalence of diabetic retinopathy (DR) is intimately linked to the upsurge in prevalence of diabetes mellitus [2]. Diabetic retinopathy (DR) is the leading cause of vision loss in adults aged 20–74 years [3]. Proliferative diabetic retinopathy (PDR) is the most common vision-threatening lesion particularly among patients with type 1 diabetes. However, diabetic macular edema (DME) is responsible for most of the visual loss experienced by patients with diabetes [4, 5]. The retinal pigment epithelium (RPE) is located between light-sensitive outer segments of the photoreceptors and vessels of the choriocapillaris. Maintenance of visual function requires close interaction between RPE and photoreceptors. RPE receives its nutrition from choriocapillaries, which in turn is supplied by ophthalmic artery (OA) through short posterior ciliary arteries. The integrity of the choroidal capillaries is regulated by RPE. Cross-sectional imaging of the biological tissues and topography of the RPE can be non-invasively and accurately evaluated on Spectral-Domain optical coherence tomography (SD-OCT) (Cirrus High Definition OCT, Carl Zeiss Meditec Inc., CA, USA) [6]. Blood flow velocity in small orbital vessels can be studied by a Color Doppler imaging using Philips Affiniti 70G Ultrasound System, Vista, California (USA). It is a non-invasive and sensitive technique to detect ocular hemodynamic changes in different stages of diabetic retinopathy. The peak systolic (PSV), end diastolic (EDV) and mean blood flow velocities over the cardiac cycle are calculated by built in software. Color Doppler imaging determines velocity of moving cells. Resistive index (RI) reflects vascular resistance peripheral to the measuring location [7]. In an earlier study [8], we discovered RPE topographic alterations, on SD-OCT RPE segmentation map, which correlated with severity of diabetic retinopathy. The present study was undertaken to evaluate the correlation of RPE alterations on SD-OCT with RI of ophthalmic artery, in diabetic retinopathy, to our knowledge, for the first time.

## Methods

The authors confirm adherence to the tenets of the Declaration of Helsinki. Study was undertaken after institutional review board clearance and a written informed voluntary consent from all the study subjects. The study was a tertiary care centre based cross sectional study. Diagnosis of type 2 diabetes mellitus was made according to American Diabetes Association (ADA) guidelines which include fasting plasma glucose level ≥ 126 mg/dl, two hour plasma glucose level ≥ 200 mg/dl during an oral glucose tolerance test [9]. Seventy five consecutive cases of diabetes mellitus in the age group of 40–70 years were taken. On the basis of clinical and imaging features, study subjects were divided into three groups based on the early treatment diabetic retinopathy study (ETDRS) classification [10]: diabetes patients without retinopathy (n = 24), non-proliferative diabetic retinopathy (n = 27), and proliferative diabetic retinopathy (n = 24). Right eye of all study subjects was included in symmetrical involvement. In asymmetrical involvement, the eye with more severe form of the disease was included. Twenty four healthy controls were also included. Subjects with age related macular degeneration, ocular or systemic diseases affecting the retinal vascular pathology, subjects with previous intravitreal injection(s), ophthalmic surgical or laser interventions, with media haze at any level giving signal strength of less than 5 on SD-OCT (Cirrus High Definition OCT, Carl Zeiss Meditec Inc., CA, USA) were excluded from the study. Patients on any medications causing change in blood flow (calcium channel blockers, pentoxifylline, statins, antiplatelet agents, anticoagulants) were also excluded.

**Fig. 1:**
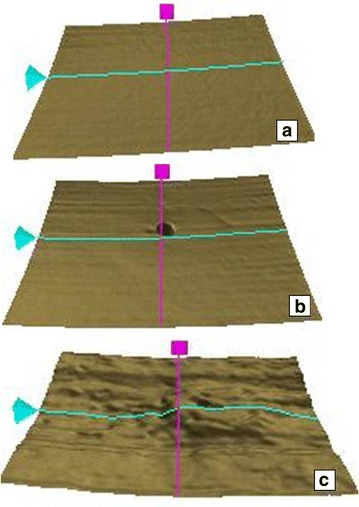
Single layer retinal pigment epithelial maps showing retinal pigment epithelial topographic alterations in diabetic retinopathy alterations of this layer was graded into 3 categories: **a** Grade 0: No RPE alterations, **b** Grade 1: focal- alteration in predominantly up to 2 quadrants of map, **c** Grade 2: global- alteration in more than 2 quadrants of map

**Fig. 2:**
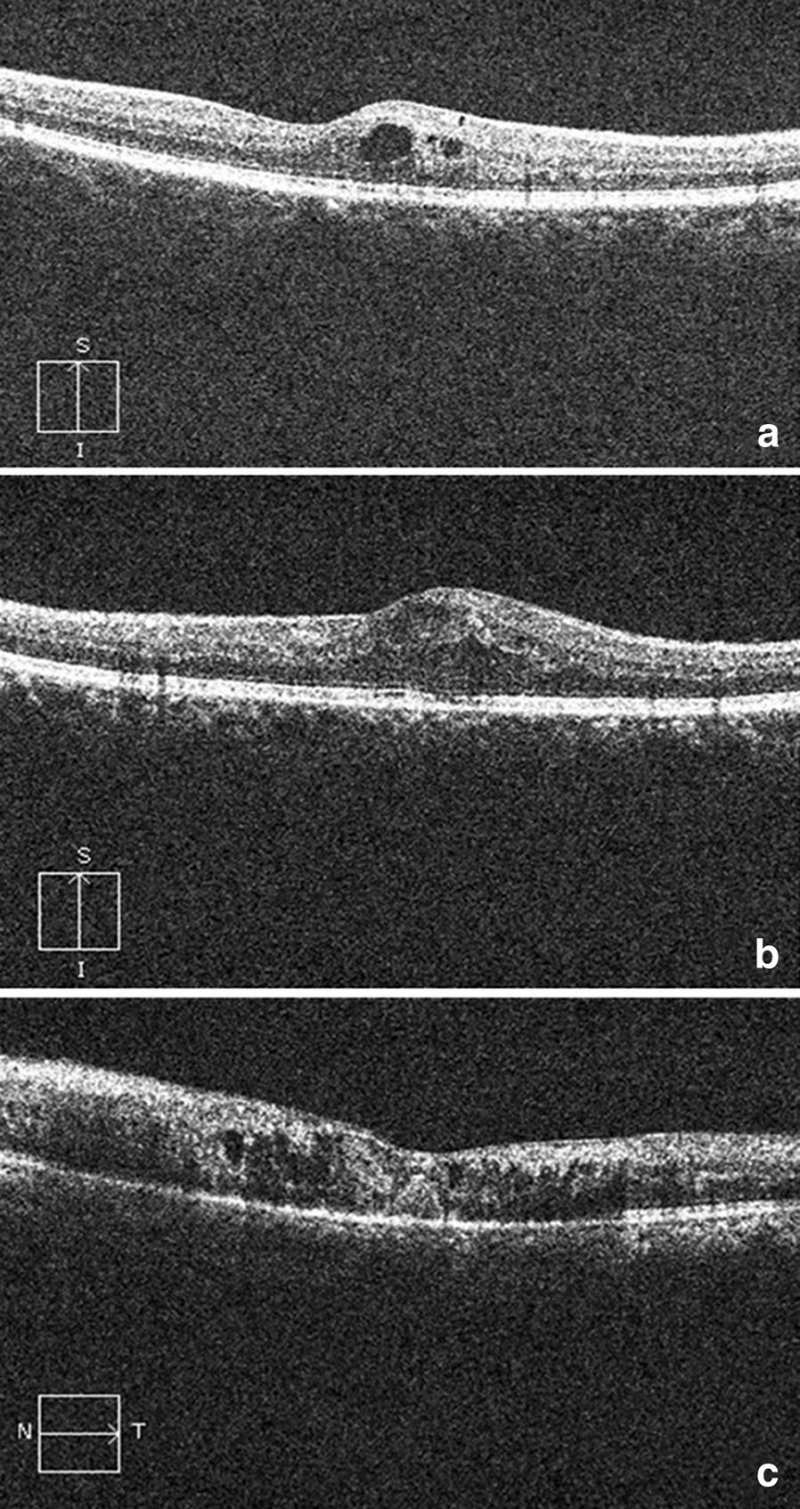
SD-OCT macular cube showing ellipsoid zone (EZ). **a** Grade 0: intact EZ, **b** Grade 1: focal disruption (localized, subfoveal EZ disruption), **c** Grade 2: global disruption (generalized EZ disruption throughout the macular cube)

The best-corrected visual acuity was documented on the logMAR scale. Patient age and gender were documented. All study subjects underwent detailed fundus evaluation using stereoscopic slit lamp biomicroscopy and indirect ophthalmoscopy. Digital fundus photography and fluorescein angiography were done using Zeiss fundus camera FF 450 Plus with pixel width of 0.0054 and image size 2588 × 1958 (Carl Zeiss Meditec AG 07740 Jena Germany). Every study subject underwent macular thickness analysis using the macular cube 512 × 128 feature of SD-OCT. RPE topography was studied on the SD-OCT segmentation map. RPE alterations were graded as [8]: grade 0, no RPE alterations; grade 1, RPE alterations in up to two quadrants; and grade 2, RPE alterations in more than two quadrants (Fig. 1). On horizontal and vertical SD-OCT scans, retinal photoreceptor ellipsoid zone disruption was graded into three categories [11], Grade 0: Intact photoreceptor ellipsoid zone, Grade 1: Focal disruption (photoreceptor ellipsoid zone disruption indicating subfoveal localized involvement, Grade 2: Global disruption (photoreceptor ellipsoid zone disruption indicating generalized involvement within the macular cube) (Fig. 2). Two experienced observers masked to the status of diabetic retinopathy assessed the grades of RPE alterations and ellipsoid zone disruption. The interobserver correlation was computed using Spearman’s rank correlation.

Blood samples were collected and glycated hemoglobin (HbA1c) was measured on autoanalyser using standard protocol. Color Doppler imaging was done using the Philips Affiniti 70G Ultrasound System, Vista, California (USA). Blood flow was studied in the ophthalmic artery (OA). Vascular resistance against blood flow was calculated by the following formula: RI = (PSV-EDV)/PSV where, RI = resistance index, PSV = peak systolic velocity and EDV = end diastolic velocity.

### Statistics

Data were summarised as Mean ± SE (standard error of the mean). Interobserver correlation for RPE alteration and EZ disruption was computed using Spearman’s rank correlation. Groups were compared by one way analysis of variance (ANOVA) and the significance of mean difference between the groups was done by Newman-Keuls test after ascertaining normality by Shapiro–Wilk’s test and homogeneity of variance between groups by Levene’s test. Categorical (discrete) groups were compared by Chi square (χ^2^) test. Pearson correlation analysis was done to assess association between the study variables. A two-tailed (*α *= 2) *p* < 0.05 was considered statistically significant. Analyses were performed on SPSS software (Windows version 17.0).

## Results

Analysis of variance showed no statistically significant difference in age among the study groups (F = 1.64, *p* = 0.183). χ^2^ test showed similar sex proportions among the study groups (χ^2^ = 2.035, *p* = 0.562). ANOVA also showed significant difference in visual acuity (F = 105.74, *p* < 0.001), S. HbA1C levels (F = 55.83, *p* < 0.001) and SD-OCT based bioimaging parameters, namely, EZ disruption and RPE topography among the study groups (Table 1). Interobserver correlation for RPE alterations was observed to be r = 0.78 (*p* = 0.001). χ^2^ test revealed significant increase in grades of RPE alterations (χ^2^ = 71.83, *p* < 0.001) and EZ disruption (χ^2^ = 60.59, *p* < 0.001) with the severity of diabetic retinopathy.

**Table 1:** Demographic, clinical, OCT, topographic and color doppler parameter levels (Mean ± SE) of four groups

Variable	Controls (n = 24) (%)	NO DR (n = 24) (%)	NPDR (n = 27) (%)	PDR (n = 24) (%)	F/χ^2^ value	*p* value
Age (years)	60.23 ± 1.34	58.68 ± 1.54	58.28 ± 2.32	63.41 ± 1.99	1.64	0.183
*Sex*						
Female	8 (36.4)	9 (40.9)	7 (28.0)	5 (22.7)	2.035	0.562
Male	14 (63.6)	13 (59.1)	18 (72.0)	17 (77.3)		
HbA1c (%)	5.35 ± 0.11	7.91 ± 0.19	8.42 ± 0.28	8.88 ± 0.18	55.83	< 0.001
BCVA	0.09 ± 0.03	0.33 ± 0.14	0.74 ± 0.07	1.19 ± 0.02	105.74	< 0.001
(log MAR)	(0.07–0.11)	(0.20–0.48)	(0.65–0.79)	(1.16–1.20)		
*EZ*						
Disruption absent	22 (100.0)	21 (95.5)	14 (56.0)	0 (0.0)	60.59	< 0.001
Disruption present	0 (0.0)	1 (4.5)	11 (44.0)	22 (100.0)		
*RPE*						
Disruption absent	22 (100.0)	22 (100.0)	20 (80.0)	0 (0.0)	71.83	< 0.001
Disruption present	0 (0.0)	0 (0.0)	5 (20.0)	22 (100.0)		
RI-OA	0.61 ± 0.01	0.79 ± 0.01	0.81 ± 0.02	1.02 ± 0.09	14.23	< 0.001

*NO DR* no diabetic retinopathy, *NPDR* non proliferative diabetic retinopathy, *PDR* proliferative diabetic retinopathy, *BCVA* best corrected visual acuity, *HBA1c* glycosylated haemoglobin, *EZ* ellipsoid zone, *RPE* retinal pigment epithelium, *RI-OA* resistive index of ophthalmic artery

**Fig. 3:**
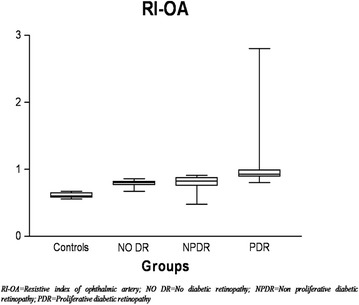
Box plot showing resistive index (RI) of ophthalmic artery (OA) of four groups

**Fig. 4:**
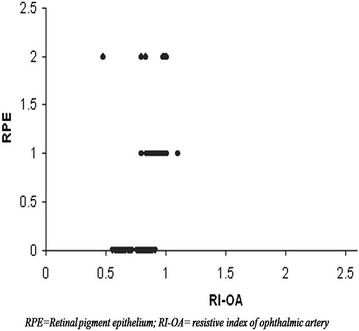
Scatter plot illustrating correlation between resistive index (RI) of ophthalmic artery (OA) and retinal pigment epithelium (RPE) alterations

**Fig. 5:**
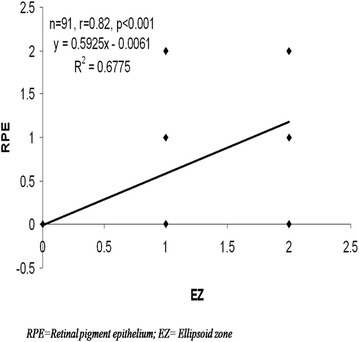
Scatter plot illustrating correlation between retinal pigment epithelium (RPE) alterations and ellipsoid zone (EZ) alterations

Color Doppler imaging based vascular resistive index was analysed in OA. ANOVA revealed a significant increase in RI of OA (F = 14.23, *p* < 0.001) with severity of diabetic retinopathy (Fig. 3). Pearson correlation analyses revealed a significant positive correlation between RI of OA with grades of RPE alterations (r = 0.48, *p* < 0.001) (Fig. 4) and also between grades of RPE alterations and EZ disruption (r = 0.82, *p* < 0.001) (Fig. 5).

## Discussion

In our present study, we found a significant increase in LogMAR BCVA, RPE topographic alterations and grades of EZ disruption with the severity of DR. We also evaluated the correlation of resistive index, a parameter of vascular resistance, in OA with grades of RPE alterations. A significant positive correlation was observed between RI of OA, with severity of diabetic retinopathy. Increase in RI of OA was found to be associated with increased grades of RPE alterations and EZ disruption on SD-OCT. Also a positive correlation was observed between grades of RPE alterations and EZ disruption. Our earlier studies highlighted positive correlation between grades of RPE alterations [8] and EZ disruption [11] with LogMAR BCVA.

Resistive index has been characterised as a marker of vascular resistance. Vascular resistance is determined by RI. Resistive index increases with increasing resistance, with vascular compliance taken into account [12]. In a study on ocular hemodynamics, Mendivil et al. [13] found a significant differences in flow velocities in OA and central retinal artery (CRA) between healthy and diabetic individuals. Ocular blood flow velocity was found to be lower in PDR patients as compared to normal. In another study, Goebel et al. [14] compared systolic, diastolic and mean velocities in CRA, OA and PCA in cases with PDR, NPDR and controls and found that perfusion velocity in CRA showed significant difference. They found a significant positive correlation between severity diabetic retinopathy and flow velocity in the CRA.

Retinal capillary endothelium damage in diabetes occurs due to basement membrane thickening, pericyte loss, increased expression of intercellular adhesion molecule-1 (ICAM-1) [15], advanced glycation end products (AGEs) [16], oxidative and nitrosative stress [8] and decreased capillary perfusion. This in turn leads to fluid leakage out of the capillaries resulting into DME, capillary closure and decreased capillary blood flow. In addition, positive correlation of AGEs with grades of RPE alterations has been observed in diabetic retinopathy [17]. Blood supply to retina is decreased due to biochemical and biomolecular changes with resultant retinal ischemia and increased vascular endothelial growth factor (VEGF) release [18–20].

Blood flow to adjacent retinal capillaries is increased due to retinal ischemia, resulting in increase in vessel wall shear stress [21]. Capillary closure and alterations in rheological properties of blood also results in increased shear stress. Locking of the vessel occurs due to increased glycation and thickening of the basement membrane [22]. Also in the presence of dilated vasculature the systemic blood pressure is more easily transmitted to the microcirculation resulting in increased capillary pressure. As a result shear stress in vessel wall increases as the vessel diameter is unable to change, leading to mechanical injury to the vascular endothelium. This circumferential stress resulting into mechanical damage to the endothelium is directly proportional to the perfusion pressure and radius and inversely proportional to the thickness of the vessel wall [23]. Hence, circumferential stress damage occurs more on vessel with larger diameter resulting into further dilatation of vessel. The tension resisting circumferential stress in the vessel wall has an inverse relationship with the radius of the vessel, as a result tension to counteract circumferential stress is not attained in a dilated vessel, and therefore there is a tendency towards dilatation with consequent hyperperfusion.

Additionally, several other factors resulting in hyperperfusion are abnormal autoregulation of the retinal circulation [24], increased conductance as an autoregulatory response to retinal ischemia [25], endothelin-1 resistance, inhibition of calcium influx channel in smooth muscle cells and increased activity of nitric oxide synthase. As these changes occur in retinal vasculature the resistive index increases. In the present study, we found an increase in RI of OA with severity of DR, grades of RPE alterations and EZ disruption.

RPE is essential for the maintenance and survival of the overlying photoreceptor cells and thereby sustains structural and functional integrity of EZ. It is also involved in regulation of the integrity of the choroidal capillaries [26, 27]. Retinal pigment epithelium is necessary for the maintenance of photoreceptor excitability by stabilizing ion composition in the subretinal space [28–30], phagocytosis of shed photoreceptor outer segments [26, 31–33] and rebuilding light-sensitive outer segments from the base of the photoreceptors. Moreover, the structural integrity of choriocapillaris endothelium and photoreceptors is maintained by a variety of growth factors secreted by RPE. These growth factors function in both endothelial cell differentiation and photoreceptor differentiation [34–38]. Thus RPE is essential for visual function and development of retinal structures. A failure of any one of these functions can lead to degeneration of the retina, loss of visual function, and blindness.

RPE cells are dependent on choroid for its metabolic needs. The present study highlighted that RI of OA increases with severity of DR. As a result, choroidal blood supply is hampered due to lack of autoregulation. Thus, an increase in RI of OA also impairs blood supply of RPE resulting in structural changes expressed as topographical alterations in RPE on SD-OCT. RPE is essential for maintenance of retinal photoreceptors integrity and function. Alterations in RPE also correlated with EZ disruption. Our earlier studies have shown negative correlation between grades of EZ disruption and visual acuity [39]. Recently, we discovered RI of OA as a sensitive bioimaging biomarker for the severity of DR [40]. It can be concluded that with increased severity of DR, an increase in RI of OA results in decrease in blood flow to RPE. This leads to RPE topographic alterations and resultant decrease in VA. Thus from our present study, it may be hypothesised that RI of OA is one of the important factors causing RPE topographical alterations.

## Abbreviations

SD-OCT: spectral domain optical coherence tomography
RPE: retinal pigment epithelium
DR: diabetic retinopathy
EZ: ellipsoid zone
DME: diabetic macular edema
OA: ophthalmic artery
RI: resistive index
